# The Impact of Anime on Children with Autism Spectrum Disorder (ASD)

**DOI:** 10.3390/children12081078

**Published:** 2025-08-17

**Authors:** Efthalia Tzila, Eleni Panagouli, Maria Tsouka, Stavroula Oikonomou, Aikaterini Koumparelou, Maria Tsolia

**Affiliations:** 1 Developmental Assessment Unit, Second Department of Pediatrics, “P. & A. Kyriakou” Children’s Hospital, School of Medicine, National and Kapodistrian University of Athens, 11527 Athens, Greecestav_ek@yahoo.gr (S.O.); mtsolia@med.uoa.gr (M.T.); 2 Department of Psychology, Université Lumière Lyon 2, 69365 Lyon, France; m.tsouka@univ-lyon2.fr

**Keywords:** autistic girls, autistic boys, anime appeal, anime consumption, anime effects, interests in autism, social interaction, emotional expression, mental health

## Abstract

Autism Spectrum Disorder (ASD) presents unique challenges in social interaction, communication and emotional regulation. Recent research has explored the potential influence of anime consumption among children with ASD, and the current findings suggest both beneficial and adverse effects. This review examines the role of anime in fostering social learning, emotional resilience, and cognitive engagement while also addressing concerns regarding its cultivation of social withdrawal, unrealistic expectations, and over-reliance on fictional narratives. By analyzing existing literature, this paper provides insights into the nuanced relationship between anime and ASD, highlighting the possibility that patterns of engagement may be associated with both positive and negative outcomes. Understanding these dynamics is crucial for parents, educators, and clinicians seeking to support the well-being and development of children with ASD.

## 1. Introduction

Autism Spectrum Disorder (ASD) constitutes a neurodevelopmental condition characterized by challenges in social interaction, communication, and repetitive behaviors [1]. While ASD is more frequently diagnosed in males, an expanding body of research underscores the distinct ways in which it manifests in females [2]. Girls with ASD often employ unique coping mechanisms, such as social masking, and exhibit different patterns of special interests compared to their male counterparts. A growing area of inquiry pertains to the relationship between boys and girls with ASD and their engagement with anime.

Anime, a Japanese animation style encompassing a broad spectrum of themes and genres, has garnered significant global popularity among neurodivergent individuals. Remarkably, that many neurodivergent viewers engage with anime as a means of regulating their emotions and sensory experiences, finding familiarity within its “strange” worlds and forming connections with others who experience the world differently. Anime thus emerges not merely as a form of artistic expression, but as a powerful tool for both personal and collective empowerment [3]. However, much of the available literature stems from online clinical observations or limited empirical studies primarily in East Asian or Western contexts, suggesting potential cultural biases. It is important to acknowledge that current conclusions about anime’s appeal are derived largely from qualitative, observational, and or self-reported data, necessitating cautious interpretation.

The appeal of anime for children with ASD may derive from its visually rich storytelling, character-driven narratives, and structured yet exaggerated social interactions. However, the implications of anime consumption on their development, social competencies, and psychological well-being remain a subject of debate. This review critically examines the potential benefits and challenges associated with anime engagement among children with ASD, emphasizing social, cognitive, and emotional dimensions [3]. Additionally, gendered differences in special interests, with girls often gravitating toward socially normative or relationally focused themes, challenge dominant diagnostic frameworks that have historically been attributed on male-centric presentations of autism [4,5]. This difference underscores the importance of examining media influences through a gendered lens, particularly since diagnostic frameworks have historically underrepresented or misunderstood the female autistic phenotype. As a result, media engagement patterns may vary significantly by gender, highlighting the need for research approaches that account for these differences to better understand and support diverse experiences.

Against this backdrop, there is a growing interest in the intersection of autism and mediated cultural forms, notably Japanese anime. Anime, a globally disseminated form of animated storytelling originating from Japan, has garnered substantial popularity among neurodivergent populations due to its highly expressive visual language, emotionally saturated narratives, and consistent character archetypes [3]. The unique narrative structures and aesthetic conventions of anime appear to resonate strongly with individuals—particularly children and adolescents—with ASD who may be drawn to its predictable emotional cues, symbolic depth, and richly layered world-building [3].

From a developmental perspective, anime’s appeal may lie in its capacity to scaffold social cognition. For children with ASD, who frequently struggle with decoding nonverbal cues and navigating unstructured social exchanges, the exaggerated facial expressions and clearly delineated emotional arcs characteristic of anime may facilitate improved emotion recognition and interpersonal understanding [6,7,8,9]. Furthermore, participation in anime-related subcultures (e.g., fan communities, cosplay, digital forums) may offer alternative spaces for social engagement, identity formation, and creative expression—domains that are often challenging for youth with ASD in conventional social settings [10,11].

However, the increasing affinity for anime among children with ASD also invites critical examination of its psychosocial implications. While engagement with animated media can support the development of empathy, narrative comprehension, and even linguistic competence—especially when subtitles are used [12]—it may also pose risks. Excessive screen time, immersion in idealized or fantastical relationships, and a tendency toward escapism can, in some cases, reinforce patterns of social withdrawal or perpetuate unrealistic expectations about real-world interactions [10,13].

Through this literature review we seek to interrogate the multifaceted role of anime consumption in the lives of boys and girls with ASD, with a particular emphasis on the differential gendered experiences within this phenomenon. We aim to synthesize current findings on the cognitive, emotional, and social outcomes associated with anime engagement and to contextualize them within broader developmental psychology, media studies, and neurodiversity research discourses [6,7,10]. Importantly, we examine the dual potential of anime—as both a therapeutic and maladaptive medium—highlighting the critical need for balanced media engagement strategies tailored to the specific needs and vulnerabilities of neurodivergent youth [8,9,13,14].

By adopting a gender-inclusive and interdisciplinary lens, we aspire, through this review, to contribute to a more nuanced understanding of how cultural consumption intersects with neurodevelopmental profiles. Such insight is particularly salient in light of the increasing digitization of childhood and the growing influence of media in shaping identity, affect regulation, and social belonging for children on the autism spectrum [5,10,12].

## 2. Materials and Methods

Τhis is a detailed literature (narrative) review on the impact of anime on social interaction, emotional regulation and cognitive assessment of boys and girls on the autism spectrum. The present literature review was developed through a structured search of academic publications from 2010 to 2025. Search terms as “Anime”, “children with ASD’’, “gender differences”, “social interaction”, “emotional expression”, “cognitive progress”, “mental health” were utilized on the electronic database “PubMed” and Google Scholar in order to identify relevant studies. A total of 2875 were retrieved, from which 23 studies were included after screening. Study selection was conducted independently by two reviewers (ET and MT), and discrepancies were resolved through discussion. The included studies are presented in Table 1. Although we did not formally assess methodological quality in this review, studies were prioritized based on peer-reviewed status and relevance to the core themes.

Inclusion criteria involved studies focusing on individuals (boys and girls) diagnosed on the autism spectrum, in the English language, and studying the effects of anime on social relationships, emotional responsiveness, and cognitive abilities. Studies concerning the general neurotypical population were excluded. The collected data addressing anime consumption’s influence on boys and girls with ASD underwent preliminary categorization and then were further analyzed until we arrived at several main categories: social interaction and communication, emotional regulation and identity formation, cognitive stimulation and creativity, mental health considerations, enhanced emotion recognition through animated stimuli, therapeutic applications of anime and animated narratives, concerns about media overconsumption and social withdrawal, recent insights into media engagement, social camouflaging, and mental health, parental interactions and early social skills, a balanced approach to reducing screen time, media influence and susceptibility to misinformation, social media, friendship, and anxiety.

## 3. Results

### 3.1. Effects of Anime Consumption on Girls with ASD

#### 3.1.1. Social Interaction and Communication

Difficulties in social interaction represent a core challenge for individuals with ASD [5]. Anime offers structured and predictable social exchanges that may assist girls with ASD in decoding emotions, facial expressions, and interpersonal dynamics. Many anime series explicitly convey character emotions through exaggerated expressions and visual cues, potentially facilitating social learning. Moreover, participation in anime-related communities—particularly within the broader anime, comics, and games (ACG) subcultures—may foster a sense of belonging and offer structured opportunities for social interaction, especially for individuals with Autism Spectrum Disorder (ASD) who often experience exclusion in conventional peer settings [6,13]. As Kuo et al., 2024 highlight, these communities serve both as spaces for personal expression and as alternative social ecosystems that reduce the pressures of normative social interaction [13]. This may be especially relevant for girls with ASD, who—as Dean et al., 2017 [6] note—often engage in socially adaptive behaviors that mask difficulties, and may thus benefit from more interest-driven, less ambiguous social environments [6]. While gender differences are highlighted, further distinctions in engagement patterns between boys and girls, beyond frequency, remain an important area for future research.

Nevertheless, excessive engagement with anime may contribute to social withdrawal, particularly if it replaces real-world interactions. Certain individuals with ASD may find solace in fictional narratives, preferring them over the unpredictability of real-life social encounters, which could inadvertently reinforce isolation rather than enhance social competencies [7].

#### 3.1.2. Emotional Regulation and Identity Formation

Anime frequently explores themes of self-discovery, resilience, and personal growth. For girls with ASD, identifying with strong or relatable characters may serve as a potentially empowering mechanism, fostering self-understanding and emotional regulation [4]. Preliminary evidence suggests that such identification can contribute to increased self-acceptance and provide guidance in navigating complex emotional landscapes.

However, an over-reliance on anime characters for emotional support may impede the development of real-world coping mechanisms. Some individuals with ASD may become excessively immersed in fantasy worlds, encountering difficulties in applying learned emotional strategies to tangible social contexts. Additionally, the idealized portrayals of relationships within anime narratives may cultivate unrealistic expectations regarding social interactions and personal connections [8]. This tendency is further complicated by the idealized portrayals of relationships often depicted in anime, which may foster unrealistic expectations about interpersonal dynamics. Georgiou et al., 2024 found that individuals with higher levels of autistic traits were more susceptible to engaging with misinformation and unrealistic narratives in simulated social media environments, suggesting potential difficulties in distinguishing fiction from reality in mediated contexts [22]. This tendency toward misapprehension may be particularly relevant for late-diagnosed women with ASD, who—as Bargiela et al., 2016 [8] report—often reflect on their past social experiences through the lens of internalized misconceptions and unrealistic relational expectations [8,22].

#### 3.1.3. Cognitive Stimulation and Creativity

Anime often features intricate plotlines, diverse cultural elements, and complex world-building, which can stimulate cognitive engagement. Anime’s layered narratives and estranged aesthetic worlds provide neurodivergent audiences—particularly autistic individuals—with opportunities for both cognitive and affective engagement. The ability to think in layers, to reinterpret narrative elements from multiple perspectives, and to navigate disconnections from the ‘real’ positions anime as more than a mere sensory experience, but rather an intellectually stimulating medium. Intricate plotlines, expansive world-building, and diverse cultural references are, therefore, central to anime’s appeal, fostering symbolic exploration, immersive narrative participation, and deeper processes of affect and identity formation [3]. Girls with ASD seem to frequently demonstrate heightened pattern recognition and deep focus on specific interests [5]. Thus they find anime intellectually enriching. This engagement may enhance analytical reasoning, narrative comprehension, and even linguistic proficiency, particularly when consuming anime in its original Japanese format with subtitles.

Furthermore, anime may serve as an inspiration for creative expression, encouraging activities such as drawing, writing, and cosplay. These creative outlets can provide a constructive means of self-expression. However, given the tendency among some individuals with ASD to become intensely focused on particular interests, excessive preoccupation with anime may, in some cases, detract from engagement in academic or other productive pursuits, potentially impacting overall cognitive and personal development [7].

#### 3.1.4. Mental Health Considerations

Girls with ASD exhibit an increased susceptibility to anxiety, depression, and low self-esteem [1]. Within this context, anime consumption may function as a coping mechanism, offering emotional solace and escapism from stressors. Narratives emphasizing perseverance and self-actualization may instill a sense of reassurance and motivation [2].

Nevertheless, the representation of mental health within anime is not always accurate or constructive. Certain series romanticize maladaptive behaviors, including extreme social withdrawal, obsessive attachments to fictional characters, and avoidance of real-world challenges. Girls with ASD who experience difficulties with emotional regulation may be particularly vulnerable to these portrayals, potentially leading to distorted perceptions of mental health and self-worth [8].

### 3.2. The Impact of Anime on Boys with ASD

According to the available literature, girls with ASD tend to be more involved with anime than boys with ASD [1,13]. However, it is equally important to look at the research concerning boys with ASD. Including findings from studies about boys helps us gain a more complete and balanced understanding of how anime influences individuals with ASD overall. This is especially relevant because gender differences can shape how anime is perceived, engaged with, and how it affects emotional and social development. By comparing these experiences, we can better identify both common benefits and unique challenges faced by boys and girls with ASD, which can inform future research and clinical practices. However, generalizability is constrained by cultural specificity, small sample sizes, and a limited focus on early vs. late adolescence.

#### 3.2.1. Enhanced Emotion Recognition Through Animated Stimuli

Research shows that boys with ASD often respond well to dynamic, animated stimuli when it comes to recognizing emotions. For instance, Yan et al. conducted a study on Chinese boys with ASD and found that animated vehicles showing real emotional facial expressions helped improve their ability to identify emotions [9]. This suggests that animation, which combines movement and clear emotional cues, might be especially effective for boys with ASD. Similarly, Eggleston et al., 2021 found that boys with ASD exhibited more individualized and varied emotional responses when exposed to animation-based biofeedback compared to neurotypical children [10]. These results indicate that animated content might provide an accessible and engaging way for boys with ASD to process and express emotions, potentially supporting their emotional development in ways traditional methods might not.

#### 3.2.2. Therapeutic Applications of Anime and Animated Narratives

Anime and other forms of animated storytelling have also been used therapeutically to improve social cognition and emotional skills among boys with ASD. For instance, Golan et al., 2010 designed animated training programs aimed at helping boys with ASD to better recognize emotions, with promising results [15]. Similarly, Fletcher-Watson et al., 2016 showed that animated social stories can help children with ASD develop prosocial behaviors, encouraging better social interactions [16]. Moreover, anime has been used in group therapy settings to promote identity formation and a sense of belonging, particularly in adolescent boys with ASD, as documented by Kuo M-H et al., 2014 [17]. This highlights anime’s potential not just as entertainment but as a culturally meaningful tool that can support emotional understanding, self-expression, and social integration in therapeutic contexts.

#### 3.2.3. Concerns About Media Overconsumption and Social Withdrawal

Despite these benefits, there are significant concerns about the potential negative consequences of extensive media use among boys with ASD. Studies conducted by Mazurek et al., 2013 and Chonchaiya et al., 2011 indicate that boys with ASD tend to spend a lot of time on screens, engaged in activities such as watching anime, playing video games, and using social media [18,19]. While this can provide comfort and a way to cope with social difficulties, it may also lead to increased social withdrawal by reducing opportunities for face-to-face interactions. This pattern is similar to what has been observed in girls with ASD. Therefore, it is important to monitor and balance media use to prevent negative impacts on social development, while still recognizing the positive role that media can play.

### 3.3. Recent Insights into Media Engagement, Social Camouflaging, and Mental Health

A recent study has explored the complex relationships between media engagement, social camouflaging, and mental health outcomes in people with ASD. Kuo et al., 2024 conducted a study on creators involved in anime, comics, and games (ACG) who self-identified with high levels of ASD traits [13]. They found links between intense media engagement, efforts to mask characteristics associated with ASD (a process called social camouflaging), and various mental health challenges [13]. Although the study included participants of all genders, it highlights how anime and related media can be both a source of identity and belonging but also a coping mechanism that may sometimes lead to stress or psychological difficulties. This complexity underscores the need to carefully consider how individuals with ASD use media and how it fits into their broader emotional and social experiences.

### 3.4. Parental Interactions and Early Social Skills

Emerging evidence is beginning to place greater emphasis on the role of parental involvement in supporting the social development of children with Autism Spectrum Disorder (ASD). Specifically, one study examined how both dyadic (parent–child)and triadic (parent–child–object)interactions affected preschool-aged boys with ASD and found that these interactions had a significant positive impact on early social skill acquisition [20]. This suggests that while animated content like anime can be engaging and potentially therapeutic, its effectiveness might be enhanced when paired with meaningful real-life social exchanges.

### 3.5. Reducing Screen Time: A Balanced Approach

The topic of media overuse is particularly relevant in the context of ASD, where structured routines and screen-based interests often intersect. In a recent pilot study, efforts to reduce screen time while simultaneously encouraging social interaction led to improvements not only in children’s core ASD-related symptoms but also in levels of parental stress [21].

### 3.6. Media Influence and Susceptibility to Misinformation

Ongoing research into media use among individuals with ASD has highlighted emerging concerns about the digital environments they navigate. One recent study found that individuals with high levels of ASD traits may be more vulnerable to misinformation and conspiracy theories, particularly within online platforms that mimic social media contexts [22].

### 3.7. Social Media, Friendship, and Anxiety

Another compelling area is how anime-related digital spaces may support social connectivity. For adolescents with ASD, online communities—including anime fandoms—can offer a form of social inclusion that is less demanding than face-to-face interaction. A study conducted by Van Schalkwyk et al., 2017 showed that adolescents with lower anxiety experienced better friendship quality when engaged with social media [23].

## 4. Discussion

This review highlights the effect of anime on children with Autism Spectrum Disorder (ASD), involving a complex interaction of cognitive, social, and emotional factors. The ability of anime to promote social contact and communication is one of its main advantages. Girls with ASD can better understand facial expressions and interpersonal dynamics thanks to anime’s structured narratives and exaggerated emotional responses. Engaging in anime fandom communities can also help people feel like they belong [6]. Given the difficulty of people with more severe ASD traits in identifying emotions, anime may act as a facilitator since their response to emotional signaling improves with exposure to stimuli such as cartoons [11]. Instead of fostering the development of real-world skills, an over-reliance on anime-based interactions may lead to social withdrawal [6]. After all, girls with ASD might simply resort to it as a means for entertainment and leisure rather than for the growth of their social skills [12]. Despite promising insights, most reviewed studies suffer from methodological limitations, including small sample sizes, cross-sectional designs, and a lack of longitudinal follow-up. Furthermore, cultural homogeneity in many samples may obscure cross-cultural variations in media interpretation and use.

On the other hand, anime also contributes to emotional control and identity formation. Characters that deal with self-discovery and resilience are relatable to many girls with ASD, which helps increase confidence and self-awareness [4]. Over-reliance on fictitious relationships, however, might create false expectations for social interactions and hinder real-world coping methods [8]. Regarding the cognitive perspective, anime offers complex storylines and cultural depth, which enhance analytical thinking, language skills and creativity. Drawing, writing, and cosplay are examples of anime-related activities that can be effective means of expressing oneself [5]. However, an overemphasis on anime could detract from academic or other personal development [7].

In terms of mental health, anime can help girls with ASD cope with stress and anxiety by offering solace and a form of escape [1]. However, some anime representations could romanticize maladaptive behaviors or social disengagement, which could reinforce unhealthy coping mechanisms [8].

In any case, the consumption of anime in girls with ASD needs to be moderated, as it can certainly improve social skills, cognitive engagement, and emotional resilience, but there is a risk of it becoming problematic, reinforcing negative aspects such as social anxiety [14]. For this reason, future studies should explore the long-term potential of anime, maximizing its benefits and minimizing associated risks.

Regarding the boys, the impact of anime on boys with Autism Spectrum Disorder (ASD) reveals a complex interplay of benefits and risks, particularly concerning emotional recognition, therapeutic applications, and potential overconsumption. Anime can be a helpful tool for enhancing emotion recognition through animated stimuli, making it easier for boys with ASD to decode emotions via exaggerated expressions and visual cues [9]. Animated content has shown promise in therapeutic settings, aiding in improving social cognition and emotional skills, and promoting identity formation and a sense of belonging [15,17].

At the same time, concerns have been raised about excessive screen time among boys with ASD, which may lead to reduced real-life social interaction and increased social withdrawal [18,19]. While anime can serve as a comforting escape, overreliance on media may interfere with the development of interpersonal skills and contribute to anxiety, especially in the absence of real-world engagement [13].

Parental involvement appears to be a key moderating factor in mitigating these risks. Studies indicate that positive parent–child interactions can enhance the effectiveness of media engagement, particularly in early development [20].

Studies emphasize that combining anime-based activities with meaningful social interactions can enhance early social skills and support healthier emotional development [23]. A gender-inclusive perspective on media engagement is essential for future interventions and research. These gender differences highlight the need for gender-sensitive media guidelines. For instance, it could be speculated that relational-themed anime engagement in girls and pairing media with emotion recognition training for boys might be helpful.

However, much of the existing evidence remains preliminary or descriptive in nature. Many of the reviewed studies rely on small samples, self-reported data, or qualitative methodologies, limiting their generalizability. Moreover, comparative or longitudinal studies are relatively scarce. As this narrative review draws on heterogeneous sources, future research should prioritize more rigorous empirical designs—such as randomized controlled trials, longitudinal studies, or large-scale mixed-methods approaches—to clarify causal relationships and better support evidence-based interventions. Future research could, for instance, examine the differential effects of various anime genres (e.g., slice-of-life vs. action-based narratives) on emotional engagement, or test the use of short anime clips in structured interventions aimed at enhancing emotion recognition, social reciprocity, or communication in children with ASD.

### Additional Research and Perspectives

Recent studies have further explored the relationship between anime consumption and ASD, providing additional insights into its impact. Research has highlighted how anime may serve as a valuable medium for emotional expression and cognitive stimulation in neurodivergent individuals [13]. Moreover, findings suggest that engagement with anime communities can foster a sense of belonging and shared interests, particularly for children with ASD who may struggle with traditional social interactions [10]. However, caution is advised regarding potential over-reliance on anime for emotional regulation, as some narratives may encourage unrealistic expectations or reinforce social withdrawal tendencies [9].

## 5. Conclusions

The intersection between ASD and anime engagement in children presents a multifaceted dynamic, encompassing both potential advantages and risks. While anime consumption may offer opportunities for social learning, emotional resilience, and creative expression, excessive engagement poses challenges such as social isolation, unrealistic expectations, and an over-reliance on fictional narratives. A nuanced understanding of this phenomenon is imperative for parents, educators, and clinicians working with children on the autism spectrum. Encouraging a balanced approach—wherein anime serves as a complement rather than a substitute for real-world experiences—may help optimize its benefits while mitigating potential drawbacks. Given the dual nature of anime’s impact, active parental supervision combined with intentional media literacy education is essential to help children with ASD harness anime’s positive elements.

Moreover, anime engagement could potentially foster community, self-expression, and cognitive development. When guided appropriately, anime could contribute to creating positive socialization opportunities, providing a platform for neurodivergent individuals to interact with peers who share similar interests. However, excessive reliance on anime for emotional support can potentially hinder the development of essential real-world coping strategies.

Future research should further explore the long-term effects of anime consumption on boys and girls with ASD, particularly in relation to identity development and emotional regulation. Additionally, examining how different genres and themes within anime impact neurodivergent individuals could provide more tailored recommendations for parents and professionals. By fostering a balanced and mindful engagement with anime, it is possible to maximize its positive aspects while addressing any potential risks. Researchers should also consider the impact of anime narratives on mental health perceptions, as well as the potential of anime as a therapeutic tool in structured intervention programs for individuals on the spectrum.

Ultimately, the role of anime in the lives of children with ASD is complex and multifaceted. While its potential benefits are evident, its drawbacks must be acknowledged and managed thoughtfully. A collaborative approach, incorporating insights from psychology, education, and media studies, is necessary to fully understand how anime consumption influences cognitive and emotional development in neurodivergent individuals. By embracing a balanced perspective, professionals and caregivers can ensure that anime engagement can serve as a constructive tool rather than a hindrance to social and personal growth.

While boys and girls with ASD may engage with anime differently and experience some gender-specific effects, both groups are likely to benefit from the cognitive and emotional value that animated content can offer. Anime could potentially support emotion recognition, social skill development, and identity formation for individuals with ASD across genders. At the same time, risks like overconsumption of media and potential misinterpretation of emotions are common challenges. Because of this, it is important to promote a balanced and mindful approach to anime consumption, with guidance tailored to individual needs. Such an approach can help maximize the possible positive impact of anime both in therapeutic settings and everyday life for people with ASD.

Future interdisciplinary research is suggested to prioritize mixed-methods designs, integrating both qualitative and quantitative approaches to capture the nuanced impacts of anime on children with ASD. Longitudinal studies are essential to understand the long-term effects on identity, emotional development, and real-world social functioning. Furthermore, cross-cultural comparisons will help account for the global nature of anime consumption and its differing social meanings. Collaboration between clinicians, educators, psychologists, and media scholars is recommended to develop evidence-based media guidelines tailored to neurodivergent youth.

## Figures and Tables

**Table 1:** Categorization of Reviewed Studies.

Study No.	Author(s)	Main Focus Category	Subthemes
1	Hiller et al., 2014 [1]	Cognitive	Diagnostic criteria; gender differences
2	Kreiser & White 2014 [2]	Cognitive	Diagnostic disparities; gendered presentation skepticism
3	Rose et al., 2024 [3]	Social/Emotional	Anime’s appeal to autistic individuals; identity and sensory affinity
4	Cridland et al., 2014 [4]	Social	Adolescent female experience
5	Sutherland et al., 2017 [5]	Social	Gender differences in daily functioning
6	Dean et al., 2017 [6]	Social	Gendered behavioral camouflage
7	Tierney et al., 2016 [7]	Social	Coping strategies; social masking
8	Bargiela et al., 2016 [8]	Social/Emotional	Late diagnosis; female autism phenotype
9	Yan et al., 2018 [9]	Emotional	Emotion recognition intervention
10	Eggleston et al., 2021 [10]	Emotional/Cognitive	Biofeedback responsiveness
11	Atherton & Cross 2021 [11]	Cognitive/Emotional	Emotion recognition in eyes
12	Alhujaili et al., 2022 [12]	Social/Cognitive	Social media use differences
13	Kuo et al., 2024 [13]	Emotional/Cognitive	Camouflaging and mental health
14	Cardillo et al., 2025 [14]	Emotional	Social anxiety and media addiction
15	Golan et al., 2010 [15]	Emotional	Emotion recognition training
16	Fletcher-Watson et al., 2016 [16]	Social	Communication via iPad
17	Kuo et al., 2014 [17]	Cognitive	Media use patterns
18	Chonchaiya et al., 2011 [18]	Cognitive	TV viewing differences
19	Mazurek & Wenstrup 2013 [19]	Social/Cognitive	Sibling comparisons in media use
20	Oppenheim et al., 2025 [20]	Social	Social skills and parental interaction
21	Heffler et al., 2022 [21]	Social	Screen time and engagement
22	Georgiou et al., 2024 [22]	Cognitive	Misinformation susceptibility
23	van Schalkwyk et al., 2017 [23]	Social/Emotional	Friendship quality, anxiety

Table Legend: Social: Interpersonal interaction, friendships, social media use, masking/camouflaging. Emotional: Emotion recognition, anxiety, mental health outcomes. Cognitive: Diagnostic interpretation, media comprehension, misinformation engagement.

## Data Availability

No new data were created or analyzed in this study.

## Abbreviations

The following abbreviations are used in this manuscript:

ASD	Autism Spectrum Disorder
